# Elevated inflammatory cytokines in aqueous cytokine profile in HIV-1 infected patients with cataracts in Uganda

**DOI:** 10.1186/s12886-018-0680-y

**Published:** 2018-01-19

**Authors:** Juliet Otiti-Sengeri, Robert Colebunders, Steven J. Reynolds, Musa Muwonge, Getrude Nakigozi, Valerian Kiggundu, Fred Nalugoda, Damalie Nakanjako

**Affiliations:** 1 Makerere University College of Health Sciences, Kampala, Uganda; 2 Institute of Tropical Medicine, Antwerp, Belgium; 3 University of Antwerp, Antwerp, Belgium; 4 Division of Intramural Research, National Institute of Allergy and Infectious Diseases, National Institutes of Health, Bethesda, MD USA; 5 Johns Hopkins University School of Medicine, Baltimore, MD USA; 6 Rakai Health Service Programme, Rakai, Uganda; 7 Infectious Diseases Institute, Makerere University College of Health Sciences, Kampala, Uganda; 8 School of Medicine Department of Ophthalmology, Makerere University College of Heath sciences, P. O. Box 7072, Kampala, Uganda

**Keywords:** Cytokine profile, Aqueous fluid, Cataracts, HIV, Uganda

## Abstract

**Background:**

Cataracts occur earlier among HIV-infected adults and this is attributed to various intraocular inflammatory processes that result in early degeneration. In this study we purposed to investigate whether HIV infected individuals with cataracts develop heightened intraocular inflammatory processes compared to their HIV negative counterparts by determining the concentration of 8 cytokines in the aqueous humour of HIV-positive adults with cataracts and their HIV-negative counterparts.

**Methods:**

A cross-sectional study was conducted among consecutive adults with cataracts that were operated in an ophthalmology surgical camp in western Uganda. We determined levels of Granulocyte macrophage stimulating factor (GM-CSF), interleukin 6 (IL-6), interleukin 8 (IL-8), tumour necrotic factor alpha (TNF-a), interferon gamma (IFN-g), interleukin 4 (IL-4), interleukin 2 (IL-2), and interleukin (IL-10) in the aqueous fluid using a multiplexed cytokine analysis.

Data was entered in the SPSS version 10 and analyzed using STATA statistical software version 7.0. Categorical and continuous variables were compared using the χ2 test, Fisher’s exact test and the Student’s *t*-test. Bonferroni correction was used to cater for multiple comparison of *p* values for the various cytokines.

**Results:**

The 50 adults that underwent cataract surgery were outdoor peasants with similar exposure hours to UV radiation. The HIV-positive patients were younger {median age 43 years (SD 11.741)} compared to the HIV -negative patients {median age 66.5 years (SD 21.4)}. The mean CD4+ T cell count of the HIV-positive patients was 161 cells /mm3, and 12(48%) had started anti-retroviral therapy (ART). Pro inflammatory cytokines, GM-CSF, IL-8 and IL-10 were significantly higher among HIV-positive individuals (*p* = 0.001, 0.030, < 0.001 respectively). HIV-positive individuals on ART also showed significantly higher levels of GM-CSF, IL-8 and IL − 10 (*p* = 0.002, 0.021, < 0.001 respectively). TNF-a and IL-4 were significantly higher among those with a CD4+ T cell count greater than 200cells/mm3 compared to those with CD4+ T cell count less than 200 cells/mm3 (*p* = 0.022, 0.032 respectively).

**Conclusion:**

Cataracts among HIV-positive adults were associated with higher intraocular inflammation relative to the healthy elderly individuals with cataracts. There is need to explore the potential role of intra-ocular anti-inflammatory agents in the management of cataracts among HIV positive patients.

## Background

Cataracts are lens opacities that occur most commonly due to age-related degeneration of the lens [1]. Various factors may influence early occurrence of cataracts including developmental abnormalities, genetics, trauma, metabolic disorders, drugs, exposure to ultra-violet (UV) rays, malnutrition, excessive smoking or use of alcohol, intraocular inflammation and HIV infection [1].

A study in Uganda on visual loss among a population of 1100 HIV infected adults showed cataracts caused visual loss in 11.6%; being the second most common cause of visual loss after refractive errors [2]. Other studies including one in a Danish population also revealed that HIV–infected individuals have a higher risk of cataract surgery compared to the general population [3, 4].

### Intraocular inflammation and cataracts

The cause of cataracts among HIV-positive patients is largely attributed to intraocular inflammatory processes due to the HIV virus [5], opportunistic infections and/ or immune reconstitution -related inflammation [6]. The long-term use of nucleoside analogue reverse transcriptase inhibitors (NRTIs) [6] coupled with premature aging associated with HIV infection may also play a role in the formation of cataracts among adults living with HIV [7, 8].

Our group recently reported higher CD4+ T-cell reactivation and reduced regulatory T-cell populations in peripheral blood of HIV-positive adults with cataracts, relative to HIV-negative adults with cataracts [9]. Several studies have documented higher levels of inflammatory cytokines in the aqueous humor in various eye diseases such as uveitis, ischaemic retinal diseases and glaucoma, than in patients with simple senile cataracts [10]. In such cases, uveal and retinal tissue biopsies have shown evidence of class I and II antigen presenting cells that are not seen in patients with senile cataracts [11]. High levels of inflammatory cytokines result in structural changes due to accumulation of oxygen free radicals, long term oxidative stress and apoptosis of the lens epithelium thus inducing cataract [12].

Little is known about intra-ocular inflammation among HIV-infected individuals with cataracts in sub-Saharan Africa. We hypothesize that chronic HIV disease is associated with heightened intra-ocular inflammatory processes that increase the life-time risk of cataracts among HIV-infected adults relative to their HIV-negative counterparts. This paper reports a cross-sectional assessment of intra-ocular cytokines in the aqueous humor of HIV-positive and HIV-negative adults that received cataract surgery at an ophthalmology surgical camp in a community cohort in rural Uganda. Using multi-plex cytokine assays, pro-inflammatory cytokines including GM-CSF, IL-6, IL-8, TNF-a, IFN-g and IL-4 were assessed because of their known association with intra-ocular inflammation [13–15] as well as the immune regulatory cytokines including IL-2, IL-4 and IL-10 were assessed because of their known role in regulation of uveitis and sympathetic ophthalmitis [14, 15].

## Methods

We conducted a cross-sectional study of consecutive adults with cataracts that were operated in an ophthalmology surgical camp at the Rakai Health Sciences Programme (RHSP) in Rakai district, south western Uganda. The surgical camp at RHSP targeted individuals from the population-based cohort study, the Rakai Community Cohort Study, consisting of both HIV-1 infected and HIV seronegative individuals. This community has been under surveillance since 1994, with regular screening for HIV and close monitoring of HIV-infected adults with or without antiretroviral therapy (ART), according to the prevailing national guidelines for ART initiation over the years.

### Study participants

Patients were included in this study if they were 18 years and above and if an ophthalmologist’s consultation had diagnosed visually significant cataracts for surgery at RHSP. Patients provided written informed consent to participate in the study, including follow-up for three months from time of cataract surgery, as part of care at RHSP. Patients were excluded for aqueous analysis if they had traumatic cataracts, glaucoma, previous ocular surgery; as determined by the ophthalmologist because these conditions can cause elevated inflammatory aqueous cytokine levels without development of cataracts. Patients with severe illnesses, or any other contraindication to cataract surgery such as suppurative conjunctival infections were also excluded. HIV testing was provided to individuals that were unaware of their HIV serostatus, as part of routine pre-operative assessment.

### Study procedures

Aqueous humor was obtained on all patients at the time of surgery for analysis. Cytokine analysis was performed on the aqueous humor of all the 50 samples collected during cataract surgery from the HIV-positive and HIV-negative groups. In this study, we describe the profile found in 50 consecutive samples (25 HIV-positive and HIV-negative individuals with cataracts). The study was approved by the Uganda Virus Research Institute Science and Ethics Committee and the Uganda National Council for Science and Technology.

### Data collection

Data related to factors associated with cataract formation was collected from all patients. Demographic data included patient’s age, sex, occupation (in relation to UV exposure), and history of tobacco or alcohol use. Relevant medical history was recorded, and clinical information was sought from their medical notes where required. The data was recorded in a structured questionnaire.

### Medical history

This included date of HIV diagnosis, the patient’s latest CD4+ T cell count, the clinical stage of HIV infection, any HIV related illnesses, history of antiretroviral therapy (ART), and history of past or current systemic illnesses that could cause uveitis or cataracts (including diabetes mellitus, rheumatoid arthritis, tuberculosis, and syphilis).

### Ophthalmic examination

A complete ocular examination was carried out prior to cataract surgery. This included documentation of the patients best corrected visual acuity using a Snellen’s chart and a Landolt’s C chart. A Haag Streit slit lamp was used to examine the anterior segment and rule out signs of uveitis. Intraocular pressure was measured on all patients before surgery using an applanation tonometer. The type of cataract was assessed and recorded. Pupil function was tested and presence of synechiae noted. Direct and indirect ophthalmoscopes were used to assess the fundus where accessible. Ultrasound ‘B’scan was performed if the fundus was not accessible to assess the status of the posterior segment. Patients whose fundus was not accessible preoperatively had a post-operative fundus exam to rule out preexisting ocular diseases like retinal vein occlusion, retinal artery occlusion, diabetic retinopathy, age-related macular degeneration, and other forms of chorioretinitis. All patients had their axial length measured and biometry performed to determine the power of intraocular lens required for implantation. All patients were followed up and managed appropriately post operatively on day 1, after 2 weeks, 6 weeks and 3 months.

### Aqueous humor collection

Aqueous humor was collected under sterile conditions before commencement of cataract surgery. 100 μ liters of aqueous humor was withdrawn using a 30-gauge needle on a tuberculin syringe via an anterior chamber paracentesis. The aqueous humor sample was frozen at − 80 degrees Celsius within 2 h of collection until analysis was performed.

### Cytokine analysis

Bio-Plex Pro™ Human Cytokine, 8-plex assay was used to measure the following cytokines; IL-2, IL-4, IL-6, IL-8, IL-10, GM-CSF, IFN gamma and TNF alpha. Briefly, 50ul of the magnetic beads were added to the pre-coated wells and washed twice. Samples and standards (50ul each) were added to the respective wells and incubated for two hours at room temperature with shaking at 850 rpm (rpm). Following the incubation, the plate was washed thrice and 25ul of detection antibody was added to each well. The plate was incubated for one hour at room temperature with shaking at 850 rpm. Fifty micro-liters of streptavidin-PE was added to each well following a wash and the plate was further incubated for 10 min at room temperature with shaking. After a final wash, 125ul of assay buffer were added to each well and the plate shaken for 30 s to re-suspend the beads. The plate was read using a MAGPIX™ and the fluorescence intensity (FI) from the immunoassay was acquired and data was analyzed using Bio-Plex manager 6.0. Concentrations that were lower than the low limit of detection were defined as non-measurable. The minimum detectable concentrations for each cytokine in this study were: IL-2; 10 pg/ml, IL-4; 1.5 pg/ml, IL-6; 1.9 pg/ml, IL-8; 2.9 pg/ml, IL-10; 3 pg/ml, GM-CSF; 60 pg/ml, IFN g; 1.1 pg/ml and TNF a; 4.8 pg/ml.

### Statistical analysis

Data was entered in the SPSS version 10 and analyzed using STATA statistical software version 7.0. Categorical variables were compared using the χ2 test or Fisher’s exact test when expected cell values were less than 5. Continuous variables were compared using Student’s *t*-test. All reported *P*-values are two sided and were considered statistically significant when the type 1 error probability was < 0.05. For analysis of cytokines, we excluded individuals with identifiable predisposing factors for intra-ocular inflammation. These included active uveitis, coexisting systemic disease including with syphilis and tuberculosis, and ocular comorbidities identified postoperatively.

Cytokine levels were described using medians and interquartile ranges. Comparisons were done using the Mann-Whitney statistical test for non-parametric variables, because cytokine levels were skewed, according to the Kolmogorov-Smirnov normality test used. Bonferroni correction was used to cater for multiple comparison and *p* values were compared against α/number of comparisons instead of α = 0.05.

## Results

### Characteristics of the study population

We studied the aqueous humor of 25 HIV positive and 25 HIV negative consecutive patients with visually significant cataracts requiring surgery. All 50 patients had a normal random blood sugar prior to surgery and 96% were peasants with similar hours of exposure to UV radiation. Tobacco and alcohol use was less frequent in the HIV infected group. Of the 25 HIV negative patients, 13 (52%) were male and the age range 18 to 85 years (median age of 66.5, STD 21.4 years, with a mean age of 59 years). Table 1 summarizes the characteristics of the study population.

**Table 1:** Demographic and clinical characteristics of individuals that had cataract surgery at RHSP, south-western Uganda

Variable	HIV-positive with cataracts *n* = 25	HIV-negative with cataracts *n* = 25	*P*-value
Mean Age in years	44.6	59	< 0.0001
Age group in years			
< 50	7 (28%)	5 (20%)	
50–69	17 (68%)	7 (28%)	
70 and above	1(4%)	13(52%)	
Gender			
Male	11(44%)	13(52%)	0.571
Female	14(56%)	12(48%)	
Peasant with outdoor work location	22(88%)	23(92%)	1.000
Cigarrete smoking (Yes)	7(28%)	14 (56%)	0.045
Alcohol consumption (Yes)	5 (20%)	16(64%)	0.004
Visual acuity			
6/18 to 6/60	6(24%)	8(32%)	0.529
Less than 6/60	19(76%)	17(68%)	
Signs of uveitis^a^ (Yes)	3(12%)	1(4%)	0.609
Co-existing systemic disease¥ (Yes)	4 (16%)	3(12%)	1.000
Ocular comorbidity^b^ (Yes)	3(12%)	1(4%)	0.609
Cataract morphology			
Milky	11(44%)	5(20%)	0.017
Cortical/nuclear	2(8%)	10(40%)	
Posterior subscapular	8(32%)	4(16%)	
Anterior subscapular	1(4%)	3(12%)	

^a^active uveitis, coexisting systemic disease including with syphilis and tuberculosis, and ^b^ocular comorbidities identified postoperatively included old retinal detachment

*IL-6* Interleukin 6, *IFN-g* Interferon –gamma, *GM-CSF* Granulocyte macrophage colony stimulating factor, *TNF-a* Tumour necrosis factor alpha, *IL-2* Interleukin 2, *IL-4* Interleukin 4, *IL-8* Interleukin 8, *IL-10* Interleukin 10, *RHSP* Rakai Health Service Programme

### Characteristics of the HIV positive patients with cataracts

Of the 25 HIV positive patients, 11 (44%) were male and the age range was 26 to 76 years (median 43 years, STD 11.741, with a mean age of 44.6 years). The mean CD4 count was 161 cells /mm3, and 11/25 (44%) had WHO clinical stage II/III while 14/25 (56%) had WHO clinical stage III/IV HIV disease. Up to 13/25 (52%) were ART naïve and the mean duration of ART (for the 12 who were on ART) was 9 months. All the patients receiving ART were taking zidovudine, lamivudine and nevirapine.

### Clinical morphology of the cataracts

Clinical assessment of the cataracts revealed that a majority, 11/25 (44%), of cataracts among HIV infected were milky and posterior subscapular 8/25 (32%); while 10/25 (40%), of cataracts among HIV-negative individuals were cortical (Table 1).

Overall, 15/50 (30%) of patients that had cataract surgery had known predisposing factors; 4 with active uveitis (3 HIV positive and 1 HIV-negative), 7 with coexisting systemic disease including with syphilis and tuberculosis (4 HIV-positive and 3 HIV-negative), and 4 with ocular comorbidities identified postoperatively included old retinal detachment (3HIV positive and 1 HIV negative), as shown in Table 1. These were excluded from the analysis of intra-ocular cytokines shown in Tables 2, 3 and 4.

**Table 2:** The proportion of patients with detectable cytokines in the aqueous of patients that had cataract surgery at RHSP, south-western Uganda

Cytokine	HIV+		HIV-		*p*-value
	Number	Percentage	Number	Percentage	
IL-6	25	(100%)	25	(100%)	–
IFN-g	17	(68%)	8	(32%)	0.037*
GM-CSF	19	(86%)	22	(92%)	0.659
TNF-a	21	(95%)	12	(50%)	0.001*
IL-2	15	(68%)	5	(21%)	0.003*
IL-4	17	(77%)	8	(33%)	0.004*
IL-8	25	(100%)	25	(100%)	–
IL-10	24	(96%)	17	(68%)	0.023*

*Significant difference in proportion of cases with detectable cytokines higher among HIV positive cases compared to HIV negative cases

*IL-6* Interleukin 6, *IFN-g* Interferon –gamma, *GM-CSF* Granulocyte macrophage colony stimulating factor, *TNF-a* Tumour necrosis factor alpha, *IL-2* Interleukin 2, *IL-4* Interleukin 4, *IL-8* Interleukin 8, *IL-10* Interleukin 10, *RHSP* Rakai Health Service Programme

**Table 3:** Cytokine levels among patients that had cataract surgery at RHSP, south-western Uganda

	HIV+		HIV -		*P*-value at α = 0.05*	*P*-value at α = 0.00625^§^
Cytokine	Median (IQR)		Median (IQR)			
IL-6	1396	[432, 3999]	727	[371, 1338]	0.180	0.180
IFN-g	753	[154, 1783]	154	[154, 1315]	0.488	0.488
GM-CSF	1850	[1632, 1948]	1399	[1037, 1681]	0.001*	0.001^§^
TNF-a	79	[42, 167]	42	[6, 122]	0.152	0.152
IL-2	78	[25 , 147]	50	[25, 111]	0.445	0.445
IL-4	9	[9, 44]	21	[9, 40]	0.887	0.887
IL-8	499	[409, 1648]	397	[291, 573]	0.030*	0.030
IL-10	243	[209, 369]	146	[72, 175]	0.000*	0.000^§^

*Significant at α = 0.05

^§^Significant at α = 0.00625 corrected with Bonferroni correction for multiple comparisons

GM-CSF, IL-8 and IL-10 were found in significantly higher levels in the HIV positive group compared to the HIV negative group

*IL-6* Interleukin 6, *IFN-g* Interferon –gamma, *GM-CSF* Granulocyte macrophage colony stimulating factor, *TNF-a* Tumour necrosis factor alpha, *IL-2* Interleukin 2, *IL-4* Interleukin 4, *IL-8* Interleukin 8, *IL-10* Interleukin 10, *RHSP* Rakai Health Service Programme

**Table 4:** Cytokine levels in categorized HIV infected individuals on ART treatment that had cataract surgery at RHSP, south-western Uganda

	On ART		Not on ART		*P*-value at α = 0.05*	*P*-value at α = 0.00625^§^
	Median (IQR)		Median (IQR)			
IL-6	3092	[540, 9366]	763	[381, 1813]	0.124	0.124
IFN-g	1410	[784, 3230]	453	[154, 753]	0.115	0.115
GM-CSF	1893	[1632, 1975]	1791	[1451, 1890]	0.002*	0.002^§^
TNF-a	95	[61, 299]	79	[6, 136]	0.255	0.255
IL-2	98	[33, 223]	64	[25, 127]	0.530	0.530
IL-4	32	[9, 77]	9	[9, 26]	0.245	0.245
IL-8	1364	[419, 2573]	443	[389, 585]	0.021*	0.021
IL-10	273	[232, 497]	215	[184, 292]	0.000*	0.000^§^

*Significant at α = 0.05

^§^Significant at α = 0.00625 corrected with Bonferroni correction for multiple comparisons

Pro-inflammatory cytokine (GM-CSF, IL-8 and IL-10) were found at significantly higher levels among HIV patients on ART

*IL-6* Interleukin 6, *IFN-g* Interferon –gamma, *GM-CSF* Granulocyte macrophage colony stimulating factor, *TNF-a* Tumour necrosis factor alpha, *IL-2* Interleukin 2, *IL-4* Interleukin 4, *IL-8* Interleukin 8, *IL 10* Interleukin 10, *IQR* Interquartile range, *RHSP* Rakai Health Service Programme, *ART* Antiretroviral therapy (zidovudine, lamivudine and nevirapine)

### Cytokine assays

Table 2 summarizes the proportion of patients with detectable levels for each cytokine studied. IFN-g, TNF-a, IL-2, IL-4, and IL-10 were significantly higher in the HIV positive group compared to the HIV negative group (*p* = 0.037, 0.001, 0.003, 0.004, 0.023 respectively).

Inflammatory cytokines (GM-CSF, IL-8 and IL-10) were significantly higher in aqueous fluid of HIV-positive compared to the HIV-negative individuals that received cataract surgery (Table 3). Patients with CD4+ T cell counts greater than 200 cells/mm3 had significantly higher levels of TNF -alpha and IL-4 compared to those with CD4+ T cell count less than 200 cells/mm3(*p* = 0.002 and 0.032 respectively).

There was no statistical difference in the cytokine levels when the categories of WHO clinical stages were compared.

## Discussion

We investigated the profile of 8 important cytokines in the aqueous humor of HIV -1 infected and HIV negative population with clinically significant cataracts requiring surgery. Overall, our results provide evidence of increased intra-ocular inflammation among HIV-infected adults with cataracts.

The HIV- positive individuals with cataracts were younger (mean age of 44.6 years, *p* value < 0.0001) relative to HIV-negative adults where majority of patients were 70 years and older. The HIV-infected adults showed higher values for inflammatory cytokines, when compared with the HIV-negative group (Table 2). However, both the HIV-infected and HIV-negative individuals had similar socio-demographic characteristics such as peasant outdoor occupation and coexisting ocular or systemic diseases that could contribute to cataract formation. The alcohol and tobacco use were significantly less in the HIV-positive individuals. The risk factors for immune dysfunction/premature aging in the HIV-positive group HIV included advanced HIV disease with low CD4+ T cell count and the duration of antiretroviral therapy for those that had already received ART.

Cataracts are known to develop with advancing age, as an age-related lens degenerative disorder [16]. In addition, cataracts occur in special situations such as congenital disorders, metabolic disorders, intraocular inflammatory and ischaemic diseases or following trauma [1, 16]. Our findings show heightened levels of inflammatory markers in aqueous fluid, and the earlier age at presentation for surgery among HIV-infected in individuals suggest that inflammation during chronic HIV diseases and the associated immune dysfunction contribute to accelerated aging processes including age-related lens degeneration and cataract development [17]. Our results are consistent with previous reports of HIV-associated premature aging including early development of cataracts has been documented among HIV infected individuals in south africa, detailed imaging of lenses in a group of HIV-positive individuals revealed signs of early nuclear sclerosis and cataract formation compared to the HIV negative counterparts in the study [7, 8].

### Intraocular inflammation among HIV-positive individuals with cataracts

We found significantly higher levels of pro-inflammatory cytokine IL-6 and IL-8 in aqueous fluid of HIV positive individuals, which suggested an ongoing inflammatory process. This is further supported by significantly higher levels of TNF-a and IL-4 found among the HIV infected individuals with CD4+ T-cell count greater than 200 cells/mm3 compared those with CD4+ T cell count less than 200 cells/mm3 (Table 5).

**Table 5:** Cytokine levels in categorized CD4 count levels among HIV infected individuals that had cataract surgery at RHSP, south-western Uganda

	CD4 < 200cells/mm3		CD4 ≥ 200cells/mm3		*P*-value at α = 0.05*	*P*-value at α = 0.00625^§^
	Median (IQR)		Median (IQR)			
IL-6	1603	[374, 3677]	1190	[625, 12,463]	0.446	0.446
IFN-g	753	[154, 1290]	1469	[304, 2898]	0.539	0.539
GM-CSF	1872	[1753, 1994]	1632	[1307, 1872]	0.107	0.107
TNF-a	79	[6, 146]	230	[95, 530]	0.022*	0.022
IL-2	66	[25, 129]	147	[46, 271]	0.343	0.343
IL-4	10	[10, 34]	90	[9, 110]	0.032*	0.032
IL-8	448	[368, 1364]	836	[447, 3439]	0.319	0.319
IL-10	232	[209, 278]	365	[250, 535]	0.109	0.109

*Significant at α = 0.05

^§^Significant at α = 0.00625 corrected with Bonferroni correction for multiple comparisons

*IL-6* Interleukin 6, *IFN-g* Interferon –gamma, *GM-CSF* Granulocyte macrophage colony stimulating factor, *TNF-a* Tumour necrosis factor alpha, *IL-2* Interleukin 2, *IL-4* Interleukin 4, *IL-8* Interleukin 8, *IL-10* Interleukin 10, *IQR* Interquartile range, *RHSP* Rakai Health Service Programme

Our findings are consistent with previous studies from similar cohorts have suggested chronic inflammation and immune activation among HIV-positive adults in Uganda, even with successful antiretroviral therapy [9], irrespective of ART regimen. A similar number of patients in both groups had evidence of coexisting systemic diseases which Noteworthy is that fact that HIV-infected individuals that presented for cataract surgery were screened for any active opportunistic infections including tuberculosis and cryptococcosis as part of routine care in the RHSP HIV treatment program. Similarly, four patients with active uveitis (three HIV-infected and one HIV-negative), and seven patients with co-existing systemic disease including positive syphilis screen and tuberculosis (four HIV-positive and three HIV-negative) were excluded from the analysis intra-ocular cytokines. Despite exclusion of active infections, the authors postulate that subclinical infections could drive intra-ocular inflammatory processes leading to degenerative cataracts. It is also likely that the significantly higher levels of both IL-6 and IL − 8 among the HIV positive individuals could be attributed to a non-specific uveitis associated with HIV infection. The upregulated inflammatory pathways during chronic HIV diseases [18, 19] likely extend to the intraocular space and accelerate the life-long risk of cataracts among HIV-infected individuals. Cytokines in the aqueous humor have been widely demonstrated to play a major role in the ocular inflammatory response and resultant pathological changes in the eye including the development of cataract [20, 21]. We therefore recommend longitudinal studies to understand biomarkers of subclinical and clinical intra-ocular inflammation among individuals with chronic HIV diseases to inform innovative strategies to prevent or delay the loss of vision due to accelerated occurrence of blinding cataracts among adults living with HIV.

Individual cytokines play different roles in ocular inflammation; and the aqueous cytokine profiles also depend on the presence and stage of active ocular and systemic disease at the time they are measured. IL-6, IL-8 and GM-CSF are important pro-inflammatory cytokines, studied herein, have been demonstrated to raise in peripheral blood systemic inflammatory conditions. In our study raised levels of IL-6 and IL-8 indicate an inflammatory response among the individuals with cataracts, more so among the HIV-infected individuals. IL-6 and IL-8 primarily cause stimulation of immunoglobulins production by B-cells and augmentation of T-lymphocyte responses [13, 21]. IL-6 plays an important role in the immediate and delayed immune response. IL-8 has been frequently documented to rise in patients in toxoplasma and viral (infectious) uveitis, and in 50% of patients with idiopathic uveitis [13]. IL-8 is not detectable in normal vitreous but is found in patients with proliferative vitreous disorders. IL-8 levels were significantly higher among the HIV positive individuals on ART as compared to those not on ART. This finding is likely due to an immune reactivation process due to ART initiation, although a prospective study would provide more insight on the pathogenesis of this phenomenon.

IL-6 is usually undetectable in the vitreous of normal eyes but has been detected in aqueous in normal eyes with cataracts [20]. Its levels have been found to be significantly increased in patients following cataract surgery and in patients with ischaemic retinal diseases such as diabetic retinopathy [20, 21]. Demonstration of reduction of IL-6 levels in aqueous has been shown to be an important marker for reduction of vascular endothelial growth factor (VEGF) in patients with diabetic retinopathy on bevacizumab (anti VGEF treatment) [21, 22]. IL-6 levels are also raised in patients with idiopathic or infectious uveitis and these levels are much higher than levels in peripheral blood suggesting that its production is local within the eye [13, 20, 22].

Increased levels of GM-CSF in aqueous have been reported in long standing uveitis and are attributed to be the cause of hypotony in end stage uveitis [14, 15]. GM-CSF levels were significantly higher among HIV-positive relative to HIV-negative adults. Among the HIV-positive patients, we found two patients who had signs of old chorioretinitis at the post-operative follow up and of these, one of them had a complicated cataract with hypotony. None of the HIV-negative patients had evidence of old chorioretinitis in post-operative follow up.

### Immune regulatory cytokines

IL − 2 and IL-4 are important immune-regulators in acute phase of inflammation. Increased levels have been documented in patients with active uveitis and sympathetic ophthalmitis [14, 15]. IL-4 levels were significantly higher among HIV positive individuals with CD4+ T-cell counts greater than 200cell/mm3 compared to those who had CD4+ T-cell counts less than 200 cells /mm3 (*p* = 0.032). IL-2 and IL-4 levels were significantly lower among patients who were HIV negative compared to the HIV positive (*p* = 0.003 and 0.004 respectively; Table 2) These levels suggest their role as immunoregulators in response to an immune response in the HIV positive group.

IL-10 has both immunosuppressive and immunostimulatory properties in inflammation. It regulates differentiation and proliferation of multiple immune cells such as T and B cells, antigen presenting cells and granulocytes, which are found in abundance in inflamed or activated uveal and retinal tissue [23, 24]. It controls the inflammatory process by suppressing the expression of pro-inflammatory cytokines like TNF-a, IFN-g [20, 24]. TNF-a levels were found significantly higher in HIV-positive individuals with CD4 + T cell counts greater than 200 cells/mm3 (Table 5). Elevated levels of IL- 10 as seen in our study suggest an attempt to control non-specific intra-ocular inflammation of a stimulated state environment in the anterior chamber among HIV-positive individuals. The suppressive role of TNF-a and IL-10 is further supported by the highest proportion of patients (95 and 96% respectively; *p* = 0.001,0.023 respectively) compared to respective levels in the HIV-positive group (50 and 68%) as seen in Table 2.

High levels of immune regulatory cytokines among HIV infected adults, more so among those with CD4+ T cell counts above 200cell/mm3, reactive host immune responses to control the ongoing aberrant inflammatory processes associated with chronic HIV disease. Therefore, attempts to control the aberrant inflammation and immune activation processes among HIV-infected adults as adjunct to ART [25], could contribute to the control of the long-term complications of persistent immune activation that include end organ damage, cardiovascular diseases and other degenerative illnesses [26].

### Cataract morphology

The morphology of the cataracts of the HIV seropositive patients supports the role of an inflammatory process in the cataract formation. The majority of the HIV sero-positive individuals (76% *n* = 19) had a milky, posterior sub capsular or complicated cataract (see Table 1), which are associated an inflammatory process in the eye, compare to the HIV negative group (36%, *n* = 9).

We generate hypotheses on immune modulatory intervention to control intra-ocular inflammation and its undesired effects on the lens, to prevent/ delay development of cataracts among individuals living with HIV/AIDS. One limitation in this study was the fact that the HIV-negative patients were generally older than the HIV- positive group, and the differences in age could contribute to varied cytokine levels observed. Further study on aqueous cytokine levels, where the two groups of patients with cataracts are matched for age would increase our understanding on the role of these cytokines in the aqueous humour of HIV-positive patients with cataracts.

More work is clearly needed to understand the pathways correlation between systemic HIV-associated inflammation and intraocular inflammation, as well as the role of immune modulants in control of intraocular and systemic inflammation to modify the risk of cataracts among individuals living with HIV in sub-Saharan Africa. Furthermore, a wider range of both peripheral blood and intraocular cytokines profiles, combined with PCR detection to rule out the presence of active infections would improve our understanding on pre-senile cataract formation in HIV seropositive patients.

With the current scale up of HIV treatment to all in need, nearly 10.3 million people are receiving ART and AIDS-related deaths decreased by 36% since 2010, in the worlds’ most-affected regions; eastern and southern Africa [27]. More individuals are living longer with HIV and its associated risk of non-communicable diseases associated with accelerated aging, including cataracts [28]. There is need to further understand that the specific drivers intra-ocular inflammation and lens degeneration, to inform targeted development of immune modulatory approaches to prevent or prolong occurance of cataracts, among other non communicable diseases, as a strategy to improve the quality of life of adults living with HIV in sub-Saharan Africa.

## Conclusion

Pro-inflammatory cytokine levels were higher in aqueous fluid of HIV-positive individuals with cataracts relative to their HIV-negative counterparts with cataracts. Majority of cataracts among HIV-positive adults occurred below 50 years relative to 70 years and above among HIV-negative individuals. There is need to explore immune modulation intervention to modify risk and management of cataracts among people living with HIV.

## Abbreviations

ART: Anti-Retroviral Therapy
CI: Confidence Interval
GM-CSF: Granulocyte-Macrophage Stimulating Factor
IFN: Interferon
IL: Interleukin
IQR: Inter Quartile Range
OR: Odds Ratio
STD: Standard Deviation
